# The Effectiveness of Mesh-Less Pectopexy in the Treatment of Vaginal Apical Prolapse—A Prospective Study

**DOI:** 10.3390/diagnostics15050526

**Published:** 2025-02-21

**Authors:** Marilena Pirtea, Ligia Bălulescu, Laurentiu Pirtea, Simona Brasoveanu, Cristina Secosan, Lavinia Balan, Flavius Olaru, Alexandru Dabica, Mădălin-Marius Margan, Dan Navolan

**Affiliations:** 1 Department of Obstetrics and Gynecology, Victor Babes University of Medicine and Pharmacy, 300041 Timisoara, Romania; marilena.pirtea@umft.ro (M.P.); pirtea.laurentiu@umft.ro (L.P.); simona.brasoveanu@umft.ro (S.B.); secosan.cristina@umft.ro (C.S.); lavinia.balan@umft.ro (L.B.); olaru.flavius@umft.ro (F.O.); alexandru.dabica@umft.ro (A.D.); navolan.dan@umft.ro (D.N.); 2 Department of Functional Sciences, Discipline of Public Health, Victor Babes University of Medicine and Pharmacy, 300041 Timisoara, Romania; margan.madalin@umft.ro

**Keywords:** laparoscopic pectopexy, vaginal apical prolapse, mesh, cystocele, pelvic floor repair

## Abstract

**Objectives:** Pelvic organ prolapse (POP) is a common condition affecting women, often requiring surgical intervention. Laparoscopic pectopexy has gained popularity, but there is ongoing debate about the efficacy and safety of mesh versus thread as fixation materials. This study aims to compare the outcomes of these two techniques, focusing on cure, recurrence and postoperative complication rates. **Methods:** A prospective analysis was conducted on patients undergoing laparoscopic pectopexy for POP. This prospective study included 78 patients diagnosed with pelvic organ prolapse stage II–IV according to the POP-Q system. Thirty-six (46.15%) underwent laparoscopic pectopexy with mesh and forty-two patients (53.84%) underwent the laparoscopic pectopexy procedure with thread. Data on cure rates, recurrence, mild asymptomatic cystocele and chronic pain were analyzed. Statistical significance was assessed using chi-squared and Fisher’s exact tests. **Results:** The cure rate was high in both group: 94.4% in the Mesh group and 100% in the thread group. Recurrence of vaginal apex prolapse occurred in 5.56% of the Mesh group, while no recurrence was observed in the thread group. Chronic pain was reported in 11.11% of the Mesh group but was absent in the thread group (*p* < 0.05). The overall rate for cystocele across all participants was 44.87% (40.48% of patients in the thread group experienced mild asymptomatic cystocele, compared to 50% in the Mesh group). No intraoperative complications were reported in either group. **Conclusions:** Thread-based laparoscopic pectopexy demonstrates equivalent or superior outcomes compared to mesh, with a high cure rate, no recurrence rate and no chronic pain. These findings support the use of thread as a safer alternative, aligning with FDA guidelines on mesh usage.

## 1. Introduction

Pelvic organ prolapse (POP) is a prevalent gynecological condition, with a reported prevalence of up to 50% based on vaginal examinations and 3–6% when assessed by symptoms [1]. Although apical vaginal prolapse is less common, accounting for 5–15% of cases, it plays a pivotal role in maintaining overall pelvic organ support [2]. With the aging population, the number of POP cases is projected to rise to 4.9 million by 2050 [3].

The management of POP varies according to the severity of the prolapse and associated symptoms. Treatment options range from conservative approaches such as observation and vaginal pessaries to various surgical techniques, including vaginal, open abdominal, laparoscopic and robotic procedures. These surgical methods may involve native tissue repair or graft augmentation and may or may not aim for uterine preservation [4].

Laparoscopic pectopexy is a surgical technique used to treat apical prolapse by suspending the vaginal apex to the pectineal ligaments. This procedure typically involves the use of synthetic mesh to provide support. However, concerns about mesh-related complications have led to interest in alternative methods that utilize native tissue repairs without mesh.

In 2007, Noe introduced laparoscopic pectopexy as a novel method for apical prolapse repair [5]. This technique involves securing a mesh to the iliopectineal ligament bilaterally [5]. Research by Cosson and colleagues demonstrated that the iliopectineal ligament is significantly stronger than the sacrospinous ligament and the arcus tendinous of the pelvic fascia [6,7]. An important advantage of laparoscopic pectopexy (LP) is its reported protective effect against the development of central and lateral cystocele [8].

Laparoscopic pectopexy with thread is an emerging alternative for the treatment of vaginal apical prolapse. This approach replaces the use of synthetic mesh with surgical sutures to anchor the vaginal apex to the iliopectineal ligament. The thread-based technique aims to provide robust support while avoiding the risks associated with mesh, such as erosion, infection and especially chronic pelvic pain. This method is particularly suitable for patients who prefer or require non-mesh solutions due to their individual risk profiles. Early studies suggest that laparoscopic pectopexy with thread may offer comparable outcomes to mesh-based repairs, though further research is necessary to establish its long-term safety and effectiveness [2,8].

Objectives: This study aims to compare the follow-up outcomes of the original laparoscopic pectopexy with the mesh-less modified procedure, both techniques will be described in detail, in terms of cure rates, recurrence and postoperative complications. The postoperative complications assessed include mild asymptomatic cystocele, pelvic pain and dyspareunia.

## 2. Materials and Methods

### 2.1. Patient Selection and Inclusion, Exclusion Criteria

This study employed a prospective cohort design, a randomized clinical trial conducted at the Department of Obstetrics and Gynecology, at the Emergency Clinical Hospital of Municipality Timisoara. The surgical procedures were performed between 1 January 2021 and 31 December 2022, with the follow-up period starting on 1 January 2023, and ending on 31 December 2024. A total of 84 patients undergoing surgical treatment for pelvic organ prolapse were included and 78 completed the follow-up process. The study population was divided into two groups: the Mesh group, comprising patients treated with mesh-assisted surgical procedures, and the No Mesh group (the thread group), consisting of patients who underwent the surgical technique using thread.

This study was conducted with the approval of the Human Ethics Committee of the Victor Babeș University of Medicine and Pharmacy, Timișoara, Romania (Approval Nr. 32/2015), adhering to ethical standards. All procedures involving human participants complied with the principles of the Helsinki Declaration (2013 revision). Written informed consent was obtained from all patients prior to participation.

The following patient parameters were evaluated: BMI, parity, menopausal status and postoperative complications. The outcomes evaluated postoperative complications, such as de novo cystocele, pelvic pain and dyspareunia. Data were collected through clinical follow-up visits and patient-reported outcome measures.

Inclusion criteria required complete operative and follow-up records and surgeries performed for symptomatic pelvic organ prolapse: women aged 40–70 years, diagnosed with stage II–IV vaginal apical prolapse, through a physical examination for prolapse quantification; no significant comorbidities that contraindicate laparoscopic surgery; and informed consent provided for participation in the study.

Exclusion criteria were patients with active pelvic infection or untreated genital tract malignancy, previous pelvic reconstructive surgery involving mesh, severe cardiopulmonary conditions precluding general anesthesia, patients with connective tissue disorders or other conditions that impair wound healing and patients with incomplete documentation or loss to follow-up postoperatively. Patients were excluded from the study if they refused surgical treatment, were deemed unsuitable for surgery or had uterine pathology necessitating hysterectomy. Additionally, patients presenting with symptomatic cystocele or SUI were excluded because comprehensive surgical treatment for these conditions would require additional procedures such as anterior colporrhaphy or transobturator tape. Including such patients would complicate the assessment of the technique’s specific impact on apical prolapse.

Indications for both procedures were similar. Patients typically presented with symptoms related to vaginal protrusion. Preoperative assessment, including examination in both standing and lying positions, was crucial to prevent over- or under-correction and provided valuable insights into tissue quality.

Both procedures were performed by the same experienced surgical team, following standardized protocols to reduce variability in surgical techniques and postoperative care.

**Figure 1:**
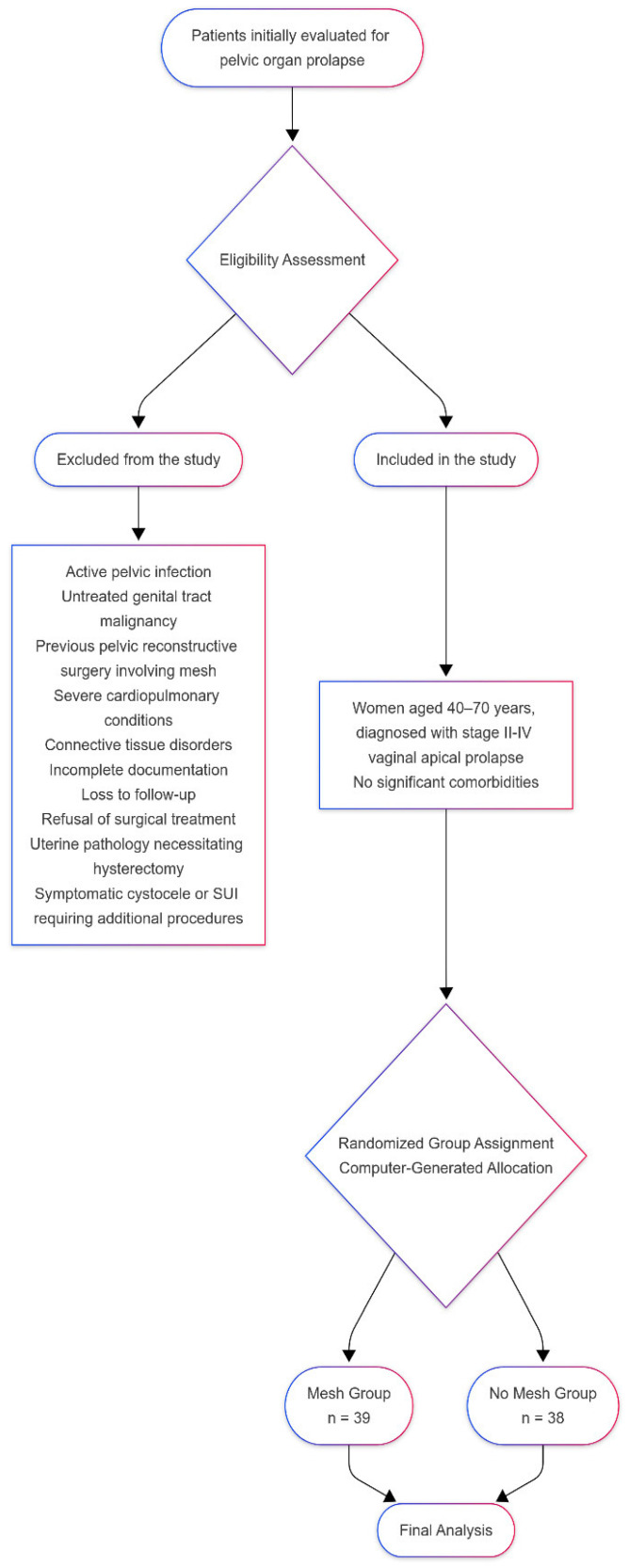
The algorithm of patient recruitment.

Patient recruitment followed a predefined algorithm (Figure 1). Outcomes were evaluated based on cure rates determined through vaginal examination. Success was defined as the cervix being positioned above the hymenal level. Validated questionnaires, including the Prolapse Quality of Life (P-QOL) and the Urinary Distress Inventory-6 (UDI-6) for patients with de novo SUI or urgency, were administered at baseline and during follow-up visits.

### 2.2. Technique Description for Laparoscopic Pectopexy with Mesh

The patient is placed in a dorsal lithotomy position under general anesthesia. Both legs are secured in adjustable stirrups to allow optimal access to the perineum and lower abdomen. A Foley catheter is inserted to empty the bladder and removed 24 h postoperatively. Bowel preparation is completed preoperatively to ensure a clear surgical field. A single prophylactic dose of antibiotic was administered preoperatively, to reduce the risk of surgical site infection.

Pneumoperitoneum was introduced using a direct entry method. An endoscopic camera was then inserted through the first 10 mm trocar, which was placed in the umbilicus. With clear visualization of the lower abdomen, three additional 5 mm laparoscopic trocars were inserted in standard positions. One 5 mm trocar was placed 5 cm below the umbilical trocar, while two 5 mm access ports were positioned 2 cm medial and above the anterior superior iliac spine on each side.

**Figure 2:**
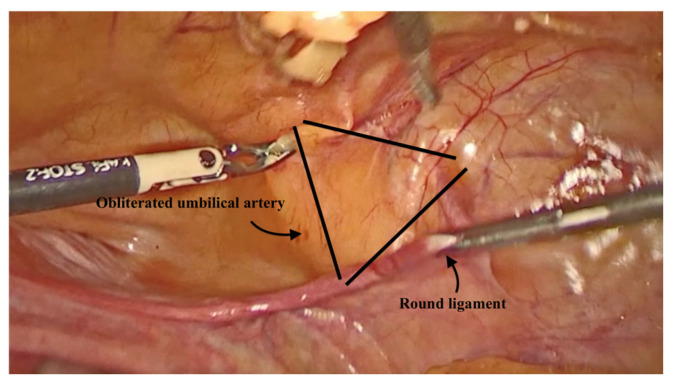
The triangular shape.

**Figure 3:**
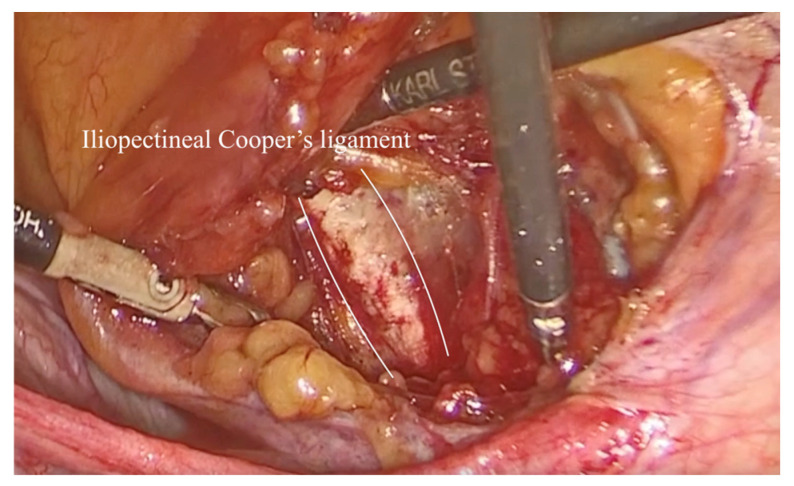
The right ileopectineal ligament of Cooper.

**Figure 4:**
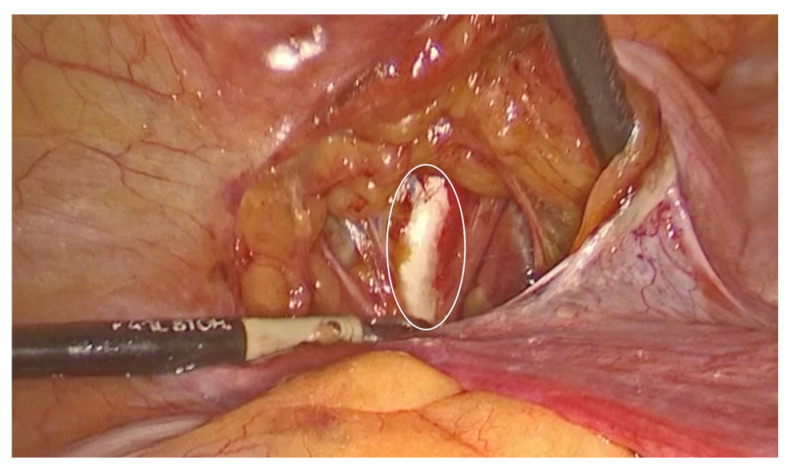
The left ileopectineal ligament of Cooper. (The circle represents the ‘The left ileopectineal ligament of Cooper’ as mentioned in the picture subtext).

To assist with the procedure, a uterine manipulator was inserted into the vagina and held in place by an assistant to expose the anterior vaginal wall. Dissection began on the left side of the pelvis. The left round ligament and the obliterated umbilical artery formed a triangular shape, around which the peritoneum was incised (Figure 2). The left iliopectineal ligament, also known as the inguinal ligament of Cooper (Figure 3 and Figure 4), was located directly under the external iliac vein and was prepared during soft tissue dissection. The same steps were followed on the right side of the pelvis. The peritoneal incisions on both sides were enlarged superficially through blunt dissection along an imaginary line between the pectineal line’s physiological axis and the anterior peritoneum of the vesico-vaginal space after the iliopectineal ligament was identified and prepared.

**Figure 5:**
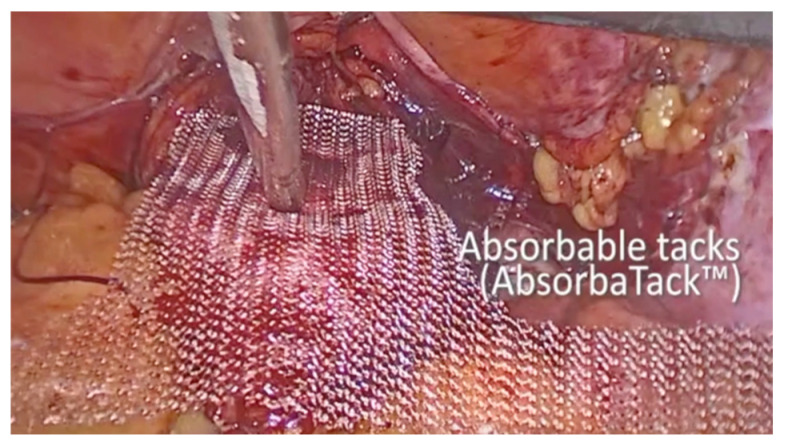
Fixation to the anterior vaginal wall.

As the procedure progressed, the vesico-vaginal space was dissected. A retractor inserted into the vagina and held by an assistant exposed the anterior vaginal wall. A T-shaped 8 cm × 15 cm polypropylene mesh was then introduced into the abdomen. Initially, absorbable tacks were used to secure the short arm of the T to the anterior vaginal wall using an AbsorbaTack fixation device (Figure 5). Next, a non-absorbable, multifilament 2.0 suture was employed to secure the lateral arms of the mesh to the iliopectineal ligaments on each side. The procedure concluded with complete closure of the peritoneum using Vicryl 2.0 sutures.

### 2.3. Technique Description for Laparoscopic Pectopexy with Thread

**Figure 6:**
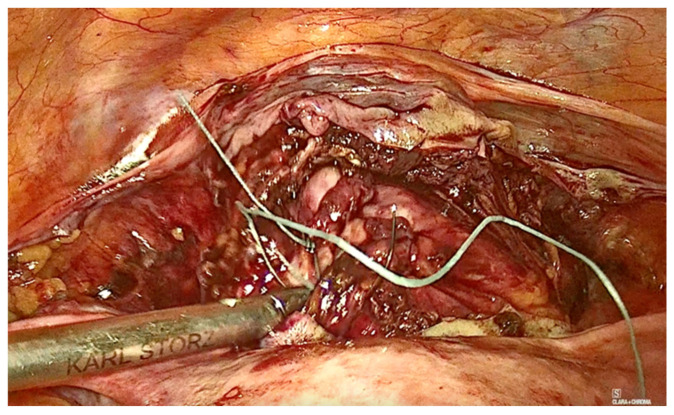
Fixation to the anterior vaginal wall.

**Figure 7:**
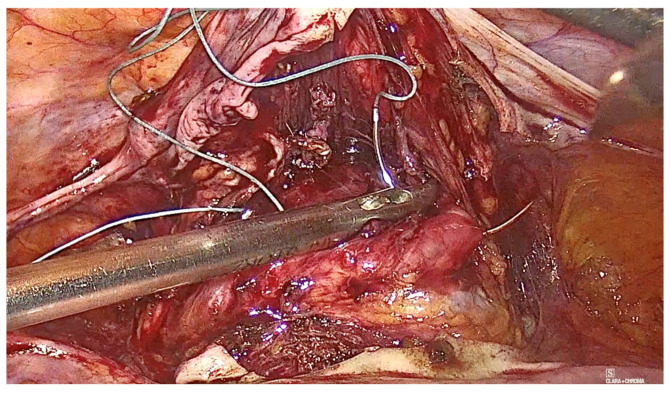
Fixation to the anterior vaginal wall.

**Figure 8:**
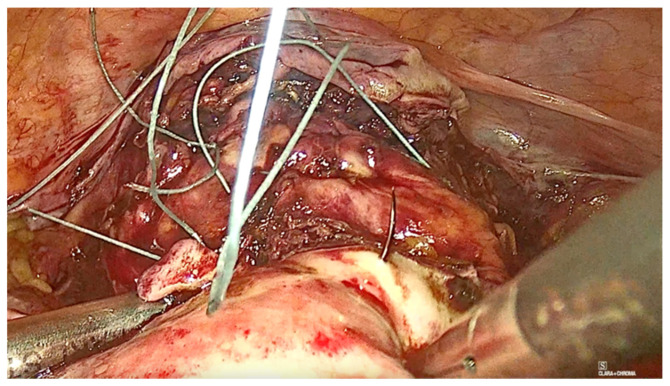
Fixation to the uterus.

**Figure 9:**
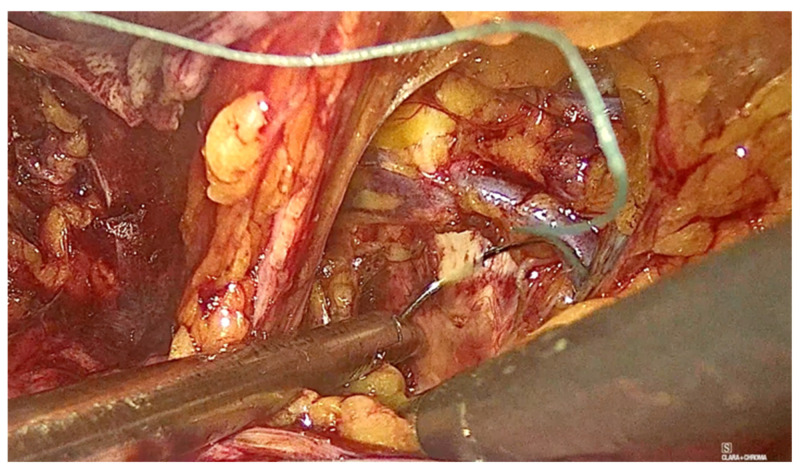
Fixation to the right iliopectineal ligament of Cooper.

**Figure 10:**
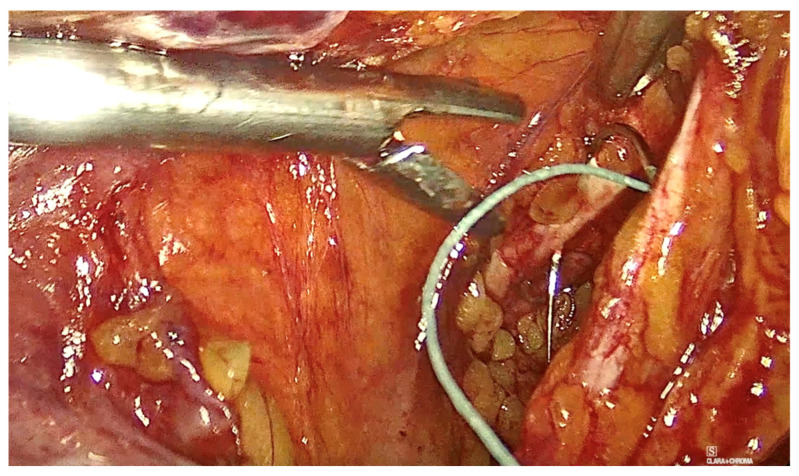
Fixation to the left iliopectineal ligament of Cooper.

**Figure 11:**
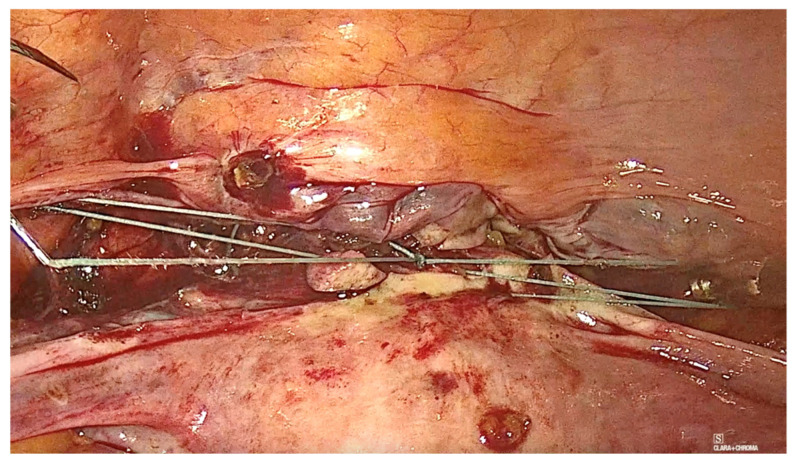
The final aspect.

**Figure 12:**
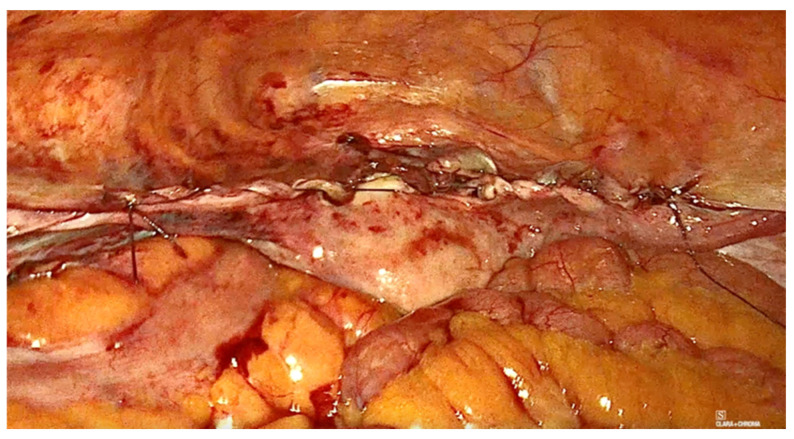
The final aspect.

The technique for laparoscopic pectopexy with thread is similar to that of laparoscopic pectopexy with mesh. However, after the dissection of Cooper’s ligament and the vesico-vaginal space, instead of using a mesh, a non-absorbable, multifilament 2.0 suture is utilized. This suture passes through the anterior vaginal wall and the uterus (Figure 6, Figure 7 and Figure 8), and is securely fixed to Cooper’s ligament (Figure 9 and Figure 10). The procedure concluded with complete closure of the peritoneum using Vicryl 2.0 sutures (Figure 11 and Figure 12).

### 2.4. Statistical Analysis

Data collection was systematic, utilizing Microsoft Excel 2021 for initial extraction and organization of demographic, clinical and surgical variables, ensuring consistency of categorical and continuous data. For statistical analysis, JASP version 0.19.2 was employed to perform subgroup analyses and variable comparisons with precision.

Statistical methods included chi-squared tests to assess the distribution of categorical outcomes, such as complete recidive, cystocele and chronic pain or dyspareunia, between the groups. Fisher’s exact test was used for variables with expected cell counts below five. For continuous variables, such as patient age and vaginal axis angle measurements, independent-sample *t*-tests were employed. Odds ratios with 95% confidence intervals were calculated to estimate the strength of associations between surgical techniques and clinical outcomes. All statistical analyses were two-tailed, with significance set at a *p*-value threshold of <0.05.

## 3. Results

**Figure 13:**
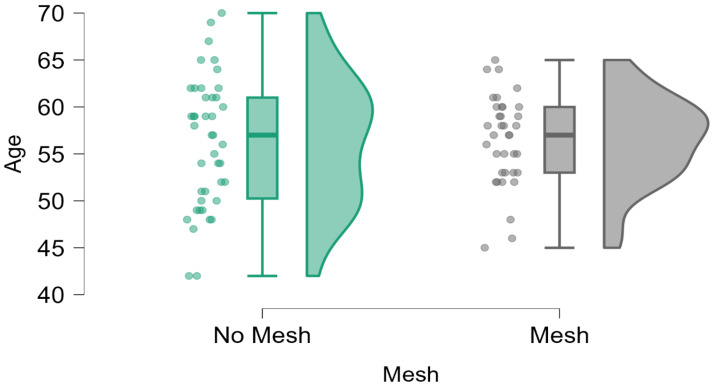
Distribution of patient age across the Mesh and No Mesh groups. Data demonstrate demographic comparability between the two groups, as indicated by similar mean and standard deviation values.

The final cohort comprised 78 patients, with a mean age of 56.30 years (SD = 5.99; range: 42–70 years). The demographic characteristics, including age and clinical history, were comparable between the Mesh and No Mesh groups, ensuring homogeneity and reducing potential confounding. A detailed distribution of age in the two subgroups is depicted in Figure 13 and Table 1, which illustrates the comparable demographic profiles across the study population.

Primary outcomes included cure rates and recurrence of apical prolapse, assessed at 1, 6, 12 and 24 months postoperatively.

The outcomes regarding the recurrence of vaginal apex prolapse demonstrated notable differences between the Mesh and No Mesh intervention groups. In the No Mesh group, there were no complete recidive cases (0%), with all patients exhibiting normal outcomes. Conversely, in the Mesh group, 5.56% of patients experienced complete recidivism, while 94.44% achieved normal outcomes. Despite this apparent difference, statistical analysis using the chi-squared test revealed that the difference was not statistically significant (Χ^2^ = 2.395, df = 1, *p* = 0.122). These findings suggest that while recurrence appears slightly more frequent with the use of mesh, the sample size and variability may limit the statistical power to detect a significant effect. The distribution of complete recidive cases by group is detailed in Table 2.

When analyzing the occurrence of mild asymptomatic cystocele, a condition commonly addressed in pelvic organ prolapse surgeries, 40.48% of patients in the No Mesh group experienced this finding, compared to 50% in the Mesh group. The slight increase associated with mesh use was not statistically significant based on the chi-squared test results (Χ^2^ = 0.711, df = 1, *p* = 0.399). Overall, 44.87% of all participants presented with mild asymptomatic cystocele. These findings underscore the importance of individualized decision-making in surgical planning, as mesh may not always provide superior outcomes for preventing mild asymptomatic cystocele. The results are summarized in Table 3, highlighting the similar cystocele outcomes across both groups.

Chronic pain or dyspareunia emerged as a critical consideration in evaluating surgical outcomes. Notably, no patients in the No Mesh group reported chronic pain or dyspareunia postoperatively (0%). In contrast, 11.11% of patients in the Mesh group experienced chronic pain. This difference was statistically significant, as demonstrated by both the chi-squared test (Χ^2^ = 4.919, df = 1, *p* = 0.027) and Fisher’s exact test (*p* = 0.041). The increased incidence of chronic pain in the Mesh group highlights a potential adverse effect of mesh use, warranting careful patient counseling regarding risks and benefits. The details of these findings are provided in Table 4, highlighting the elevated risk of chronic pain or dyspareunia associated with mesh use.

The results indicate that there are no statistically significant differences between the two techniques. However, chronic pain or dyspareunia was significantly more prevalent in the Mesh group, highlighting a critical adverse outcome of mesh use.

## 4. Discussion

The original technique of laparoscopic pectopexy involves the use of a polypropylene mesh cut in the form of an inverted T and placed to anchor the vagina and uterus at the iliopectineal ligaments. The complications related to the mesh created the context for investigating techniques that follow the same principles and anatomical landmarks but can be performed without mesh insertion. We compared the results after performing the same type of surgery using just a non-resorbable thread and the original technique that uses the polypropylene mesh. While both techniques aim to provide long-term anatomical support to the pelvic organs, they differ in terms of clinical outcomes, complications and overall effectiveness. This discussion compares the results of laparoscopic pectopexy with mesh versus thread, focusing on key outcomes such as cure rates, recurrence rates, mild asymptomatic cystocele, intraoperative complications and risk factors for recurrence.

### 4.1. Cure Rate

There are relatively few studies comparing the efficacy of laparoscopic pectopexy using mesh versus thread in the treatment of pelvic organ prolapse (POP). The cure rate is defined as the resolution of symptoms and anatomical correction. The use of mesh in pelvic organ prolapse surgeries has faced significant scrutiny due to reported complications such as chronic pain, dyspareunia, mesh erosion and infections. Thread-based laparoscopic pectopexy is a relatively newer approach that aims to avoid the complications associated with mesh. As a result, the development and study of thread-based techniques are still in their early stages, and comparative research is limited. This is because mesh-based procedures have been studied for longer periods, while thread-based techniques lack extensive long-term follow-up data. Researchers may hesitate to conduct direct comparisons without sufficient evidence on the durability and long-term outcomes of thread-based pectopexy.

Laparoscopic pectopexy using mesh has demonstrated high cure rates in the treatment of pelvic organ prolapse. For instance, S. Brasoveanu reported a cure rate of 93.59% in a group receiving laparoscopic pectopexy with mesh [9].

Additionally, a meta-analysis comparing laparoscopic pectopexy with laparoscopic sacrocolpopexy found that patients undergoing pectopexy exhibited a lower recurrence rate of prolapse post-surgery, suggesting favorable outcomes for the pectopexy approach [10].

These findings suggest that mesh-based laparoscopic pectopexy is an effective surgical option for pelvic organ prolapse, offering high cure rates and potentially lower recurrence rates compared to other surgical techniques.

The Prospective International Multicenter Pelvic Floor Study conducted by Noé et al. (2021) evaluated the short-term outcomes of combined pectopexy and native tissue repair. Their findings confirmed that pectopexy provides effective anatomical support with satisfactory functional outcomes, further validating its use in prolapse repair. These results support the idea that non-mesh approaches can achieve comparable success rates in selected patients [11].

In our study, the thread-based technique showed superior results in patients with mild to moderate prolapse, suggesting that thread can achieve similar anatomical and functional outcomes. The cure rate for the group with mesh was 94.4%, while the thread group achieved a 100% cure rate. This superior outcome in the thread group can be attributed to several factors. First, the use of a non-absorbable, multifilament 2.0 thread ensures a strong and durable fixation to Cooper’s ligament, minimizing the risk of detachment and recurrence. Unlike mesh, which relies on broader tissue integration and may be prone to foreign body reaction or erosion, the thread technique offers a more precise and localized anchoring method, reducing the likelihood of complications. These factors likely contributed to the higher cure rate observed in the thread group, particularly in patients with mild to moderate pelvic organ prolapse. Further studies with larger cohorts are needed to validate these findings and explore their long-term implications.

### 4.2. Recurrence Rate

The recurrence rate, an important indicator of long-term success, varies slightly between the two approaches. In our study, the recurrence outcomes of vaginal apex prolapse revealed significant differences between the mesh and thread groups. In the thread group, no cases of complete recurrence were observed (0%), with all patients showing normal postoperative results. In contrast, the Mesh group had a recurrence rate of 5.56%. The higher cure rate and absence of recurrence in the thread group may reflect the advantages of the thread technique in providing precise and stable anatomical support while avoiding the potential complications associated with mesh.

A prospective randomized clinical trial found an apical recurrence rate of 2.3% for laparoscopic pectopexy with mesh [5].

Factors influencing recurrence include obesity, age, parity, the vaginal axis and intra-abdominal pressure. Thread-based pectopexy may offer a slight advantage due to reduced foreign body reaction compared to mesh, which can lead to better long-term tissue integration.

### 4.3. Mild Asymptomatic Cystocele

Studies have reported varying rates of cystocele following laparoscopic pectopexy with mesh: Noe et al. conducted a prospective, randomized, comparative clinical trial and found that de novo lateral defect cystoceles were not observed after pectopexy, whereas 12.5% were found after sacropexy [5].

Astepe et al. reported a cystocele rate of 8.3% in the pectopexy group compared to 25.6% in the sacrospinous fixation group, indicating a lower incidence with pectopexy [12].

When analyzing this condition in our study, we found that mild asymptomatic cystocele rate was statistically significant based on the chi-squared test results. The overall rate for cystocele across all participants was 44.87%. (40.48% of patients in the thread group experienced mild asymptomatic cystocele, compared to 50% in the Mesh group).

### 4.4. Chronic Pain and Dyspareunia

Chronic pain and dyspareunia have emerged as key factors in assessing surgical outcomes. Notably, none of the patients in the No Mesh group reported postoperative chronic pain or dyspareunia (0%), whereas 11.11% of patients in the Mesh group experienced chronic pain. This difference was statistically significant, as confirmed by the chi-squared test. The higher incidence of chronic pain in the Mesh group underscores a potential drawback of mesh use, emphasizing the need for thorough patient counseling on the associated risks and benefits.

Several studies have examined the incidence of chronic pain and dyspareunia following pelvic organ prolapse (POP) surgeries, comparing outcomes between mesh and non-mesh interventions. A study by the American College of Obstetricians and Gynecologists (ACOG) highlights that pelvic pain, including dyspareunia, can be associated with non-exposed mesh, and such pain may not always respond to mesh removal, indicating the complexity of mesh-related complications [13].

Additionally, the literature reports that post-surgical neuropathy leading to chronic pain is a recognized complication following POP repair involving mesh. The study emphasizes the need for proper identification and treatment of post-surgical neuropathy to minimize the occurrence of chronic pain [14].

The study by Wang et al. (2022) on sacrospinous ligament fixation using an antegrade reusable suturing device (ARSD-Ney) reported significant improvements in function and quality of life while highlighting the risk of post-surgical pain. Their findings align with our study in emphasizing the importance of reducing foreign body reactions to minimize chronic pain [15].

Furthermore, another study notes that clinically significant preoperative chronic pelvic pain is a relative contraindication to the placement of permanent mesh, as pain can worsen postoperatively [16].

### 4.5. Intraoperative Complications

Common intraoperative complications may include hemorrhage, organ injury (such as bladder or bowel) and the need for conversion to laparotomy. However, these occurrences are quite rare. In a prospective, multicenter study, severe complications were observed in 1% of patients, including hemorrhage and organ damage [6].

No intraoperative complications and no early postoperative complications were reported in either group during this study. The thread’s simpler insertion and fixation technique reduces the likelihood of intraoperative injury.

### 4.6. Risk Factors for Recurrence

Obesity, age, parity and anatomical factors such intra-abdominal pressure are significant risk factors for recurrence. Both techniques show similar recurrence rates when controlling for these variables. However, thread-based pectopexy may have an advantage in obese patients, as it avoids the additional burden of a synthetic implant, which can exacerbate tissue stress. Laparoscopic procedures are beneficial for patients who are obese in terms of postoperative morbidity and wound healing [9].

Advanced age is associated with decreased tissue elasticity and muscle strength, factors that can contribute to the recurrence of POP after surgical repair [17].

A history of multiple pregnancies and vaginal births can weaken pelvic floor muscles and connective tissues, elevating the risk of prolapse recurrence [17].

High intra-abdominal pressure (due to factors like chronic constipation or obesity) can increase the risk of recurrence. Elevated intra-abdominal pressure places extra stress on the pelvic floor, which may predispose to prolapse recurrence.

### 4.7. FDA Recommendations on Mesh

The FDA has issued multiple warnings regarding the use of transvaginal mesh for POP repair due to the risk of serious complications, including mesh erosion, infection and chronic pain. As of 2019, the FDA recommended limiting the use of mesh to cases where the benefits clearly outweigh the risks and where other options have been exhausted. This has led to an increased interest in alternative methods, such as the thread-based technique, which offers comparable outcomes without the associated risks of synthetic mesh [18].

Laparoscopic pectopexy has proven to be an effective method for achieving long-lasting repairs. Successful outcomes require the surgeon to have advanced skills in intracorporeal laparoscopic suturing and knotting, along with a detailed understanding of the female pelvic anatomy.

This study is subject to certain limitations, including its reliance on a single surgical team at a single center, which may introduce bias. Furthermore, the small sample size reduces the broader applicability of the findings.

## 5. Conclusions

In summary, laparoscopic pectopexy with thread demonstrates comparable results to mesh-based techniques in terms of cure rate, recurrence rate and incidence of complications. The findings of Noé et al. support the effectiveness of pectopexy as a viable option for pelvic organ prolapse repair [11], while Wang et al. [15] and Favre-Inhofer et al. [19] reinforce the importance of assessing quality of life and long-term stability. Additionally, thread-based pectopexy aligns with FDA recommendations to minimize the use of mesh, providing a safer and equally effective alternative for the management of pelvic organ prolapse. Further large-scale, long-term studies are needed to solidify these findings and optimize patient outcomes.

## Figures and Tables

**Table 1:** Baseline characteristics of the study population, including demographic data, illustrating no significant differences in patient age between the Mesh and No Mesh groups.

	Age	
	No Mesh	Mesh
Valid	42	36
Missing	0	0
Mean	56.167	56.444
Std. Deviation	6.942	4.742
Minimum	42.000	45.000
Maximum	70.000	65.000

**Table 2:** Recurrence of vaginal apex prolapse by group. The table highlights the absence of recurrence in the No Mesh group compared to a recurrence rate of 5.56% in the Mesh group, with statistical analysis showing no significant difference (*p* = 0.122).

Mesh		Complete Recidive	Normal	Total
No Mesh	Count	0.000	42.000	42.000
	% within row	0.000%	100.000%	100.000%
	% within column	0.000%	55.263%	53.846%
Mesh	Count	2.000	34.000	36.000
	% within row	5.556%	94.444%	100.000%
	% within column	100.000%	44.737%	46.154%
Total	Count	2.000	76.000	78.000
	% within row	2.564%	97.436%	100.000%
	% within column	100.000%	100.000%	100.000%

**Table 3:** Mild asymptomatic cystocele was observed among study participants, with a slightly higher rate in the Mesh group (50%) compared to the No Mesh group (40.48%). There was no statistically significant difference between the two groups (*p* = 0.399).

		Mild Asymptomatic Cystocele		
Mesh		No	Yes	Total
No Mesh	Count	25.000	17.000	42.000
	% within row	59.524%	40.476%	100.000%
	% within column	58.140%	48.571%	53.846%
Mesh	Count	18.000	18.000	36.000
	% within row	50.000%	50.000%	100.000%
	% within column	41.860%	51.429%	46.154%
Total	Count	43.000	35.000	78.000
	% within row	55.128%	44.872%	100.000%
	% within column	100.000%	100.000%	100.000%

**Table 4:** Incidence of chronic pain or dyspareunia postoperatively. The Mesh group demonstrated a statistically significantly higher incidence (11.11%) compared to the No Mesh group (0%), as confirmed by the chi-squared and Fisher’s exact tests (*p* = 0.027 and *p* = 0.041, respectively).

		Chronic Pain/Dispareunia		
Mesh		No Pain	Chronic Pain	Total
No Mesh	Count	42.000	0.000	42.000
	% within row	100.000%	0.000%	100.000%
	% within column	56.757%	0.000%	53.846%
Mesh	Count	32.000	4.000	36.000
	% within row	88.889%	11.111%	100.000%
	% within column	43.243%	100.000%	46.154%
Total	Count	74.000	4.000	78.000
	% within row	94.872%	5.128%	100.000%
	% within column	100.000%	100.000%	100.000%

## Data Availability

Data are contained within the article.
